# Sequence-specific assignment of methyl groups from the neuronal SNARE complex using lanthanide-induced pseudocontact shifts

**DOI:** 10.1007/s10858-016-0078-1

**Published:** 2016-12-17

**Authors:** Yun-Zu Pan, Bradley Quade, Kyle D. Brewer, Monika Szabo, James D. Swarbrick, Bim Graham, Josep Rizo

**Affiliations:** 1 Department of Biophysics, University of Texas Southwestern Medical Center, Dallas, TX USA; 2 Department of Biochemistry, University of Texas Southwestern Medical Center, Dallas, TX USA; 3 Department of Pharmacology, University of Texas Southwestern Medical Center, Dallas, TX USA; 4 Monash Institute of Pharmaceutical Sciences, Monash University, Parkville, VIC Australia

**Keywords:** Methyl assignment, Pseudocontact shifts, SNAREs, Membrane fusion, Neurotransmitter release

## Abstract

Neurotransmitter release depends critically on the neuronal SNARE complex formed by syntaxin-1, SNAP-25 and synaptobrevin, as well as on other proteins such as Munc18-1, Munc13-1 and synaptotagmin-1. Although three-dimensional structures are available for these components, it is still unclear how they are assembled between the synaptic vesicle and plasma membranes to trigger fast, Ca^2+^-dependent membrane fusion. Methyl TROSY NMR experiments provide a powerful tool to study complexes between these proteins, but assignment of the methyl groups of the SNARE complex is hindered by its limited solubility. Here we report the assignment of the isoleucine, leucine, methionine and valine methyl groups of the four SNARE motifs of syntaxin-1, SNAP-25 and synaptobrevin within the SNARE complex based solely on measurements of lanthanide-induced pseudocontact shifts. Our results illustrate the power of this approach to assign protein resonances without the need of triple resonance experiments and provide an invaluable tool for future structural studies of how the SNARE complex binds to other components of the release machinery.

**Electronic supplementary material:**

The online version of this article (doi:10.1007/s10858-016-0078-1) contains supplementary material, which is available to authorized users.

## Introduction

The development of methyl-TROSY techniques using highly deuterated ^13^CH_3_-labeled (^2^H,^13^CH_3_-labeled) proteins (Tugarinov et al. 2004) has greatly facilitated the application of NMR spectroscopy to analyze the structure and dynamics of macromolecular protein complexes, thus helping to elucidate how they mediate biological processes (Rosenzweig and Kay 2014). These studies are normally more informative when sequence-specific resonance assignments of methyl groups are available. Such assignments can be obtained by triple resonance experiments for well-behaved proteins [e.g. (Tugarinov and Kay 2003)], but application of these techniques is severely hindered for proteins that are very large, have low solubility and/or are unstable. Since ^1^H-^13^C HMQC spectra of ^2^H,^13^CH_3_-labeled-proteins offers high sensitivity even for species in the 1 MDa range (Sprangers and Kay 2007), methyl resonance assignments can be obtained for large systems using these spectra in combination with systematic mutagenesis (Amero et al. 2011). However, this method requires preparation of a large number of mutant proteins, which may have a prohibitive cost. Measurements of lanthanide-induced pseudocontact shifts (PCSs) provide an alternative that can yield methyl resonance assignments for proteins of known structure without the need to obtain backbone assignments (John et al. 2007; Skinner et al. 2013). This approach remains relatively untested, but it is highly promising for large systems because it can be applied using highly sensitive 2D NMR spectra such as ^1^H-^13^C HMQC. Moreover, methods based on PCS measurements (Otting 2010; Clore and Iwahara 2009; Hass and Ubbink 2014) provide a powerful tool to determine the structures of large protein complexes in solution by NMR spectroscopy, and key work required for these approaches involves the labeling of surface-exposed residues with tags containing paramagnetic lanthanides, which can be used at the same time for methyl resonance assignment.

A particularly attractive system to apply this emerging NMR methodology is the machinery that controls neurotransmitter release by synaptic vesicle exocytosis, a process that is crucial for interneuronal communication (Sudhof 2013). Much has been learned about the release machinery (Sudhof and Rothman 2009; Jahn and Fasshauer 2012; Brunger et al. 2015; Rizo and Xu 2015), including the well-established notions that the neuronal SNAREs syntaxin-1, synaptobrevin and SNAP-25 play a central role in fusing the vesicle and plasma membranes by forming a tight four-helix bundle called the SNARE complex (Sollner et al. 1993; Hanson et al. 1997; Poirier et al. 1998; Sutton et al. 1998), that Munc18-1 and Munc13s orchestrate SNARE complex assembly (Ma et al. 2011, 2013), and that synaptotagmin-1 acts as the Ca^2+^ sensor in fast neurotransmitter release (Fernandez-Chacon et al. 2001), likely by bringing the two membranes together in a Ca^2+^-dependent manner (Arac et al. 2006). However, the mechanism by which these proteins work together to induce fusion is still unclear (Rizo and Xu 2015), in part because it has been difficult to obtain structures of the SNARE complex bound to synaptotagmin-1, Munc18-1 or Munc13-1. Such difficulties arise because the underlying interactions are of moderate affinity (Dulubova et al. 2007; Guan et al. 2008; Brewer et al. 2015) and may be influenced by the membranes, hindering crystallization.

Application of traditional NMR methods to analyze these presynaptic complexes has been challenging. Full resonance assignments and solution NMR structures are available for the two C_2_ domains (16–19 kDa) that form most of the cytoplasmic region of synaptotagmin-1 and for the syntaxin-1 N-terminal H_abc_ domain (Shao et al. 1997, 1998; Fernandez et al. 2001, 1998), and the backbone resonances of the SNARE complex four-helix bundle (32 kDa) were also described (Chen et al. 2002, 2005). With the help of these assignments, we were able to analyze a complex between the synaptotagmin-1 C_2_ domains and the SNARE four-helix bundle using lanthanide-induced PCSs, revealing a dynamic structure that nicely fits with a model whereby synaptotagmin-1 and the SNAREs cooperate to bring the membranes together to induce membrane fusion (Brewer et al. 2015). However, a recent crystal structure obtained also with soluble fragments yielded completely different syntaptotagmin-1-SNARE complex binding modes (Zhou et al. 2015). These results emphasize the need to study these interactions in the presence of membranes, which would be greatly facilitated if assignments of the methyl groups of the SNARE complex were available. However, application of triple resonance experiments is hindered by the limited solubility of the SNARE complex and the broad resonances arising in part from its elongated shape (Chen et al. 2002). NMR studies of Munc18-1 (68 kDa) and the Munc13-1 MUN domain (73 kDa), which is critical for the function of this protein (Basu et al. 2005), are also difficult because of their low solubility (less than 30 µM under physiological conditions), but note that high-quality ^1^H-^13^C HMQC spectra of ^2^H,^13^CH_3_-labeled samples of these proteins can be obtained well below these concentrations [(Ma et al. 2011) and our unpublished results] and their X-ray structures have been solved (Misura et al. 2000; Bracher et al. 2000; Yang et al. 2015).

These observations indicate that strategies involving a combination of methyl-TROSY NMR with measurements of PCSs provide viable avenues to elucidate the structures of presynaptic complexes that could yield fundamental insights into the mechanism of neurotransmitter release, but methyl resonance assignments are required for these strategies. We have first focused on the SNARE four-helix bundle because of its central functional importance. Here we report the assignment of the Ile-δ1, Leu, Met and Val methyl groups of the SNARE four-helix bundle based on measurements of PCSs induced by Dy^3+^-loaded tags placed at three different positions and a Yb^3+^-loaded tag placed at one of these positions.

## Materials and methods

### Protein expression and purification

The expression and purification of fragments spanning the SNARE motifs of rat synaptobrevin 2 (residues 29–93), rat syntaxin-1A (residues 191–253), and rat SNAP-25A (residues 11–82 and 141–203) from a PET-duet vector were previously described (Chen et al. 2002; Xu et al. 2013). Constructs to express single-cysteine SNARE mutants have also been described (Brewer et al. 2015). Unlabeled proteins were expressed in *Escherichia coli* BL21(DE3) cells in LB broth. Perdeuterated proteins were produced using M9 expression media in 99.9% D_2_O with ^2^H,^12^C-glucose as the sole carbon source (3 g/L) and ^15^NH_4_Cl as the sole nitrogen source (1 g/L). ILVM methyl-labeling was achieved by adding [3,3-^2^H_2_] ^13^C-methyl alpha-ketobutyric acid (80 mg/L), [3-^2^H] ^13^C-dimethyl alpha-ketoisovaleric acid (80 mg/L), and ^13^C-methyl methionine (250 mg/L) (Cambridge Isotope Laboratories) to the cell cultures 30 min prior to Isopropyl β-D-1-thiogalactopyranoside (IPTG) induction.

### Paramagnetic labeling and SNARE complex formation

The syntaxin-1, SNAP-25 and synaptobrevin SNARE motifs do not contain native cysteines. To label the SNARE complex with a paramagnetic tag, we obtained diverse single cysteine mutants of the SNARE motifs. To label SNARE cysteine mutants with paramagnetic tags, the protein was treated with 10 mM DTT, which was subsequently removed by gel filtration chromatography on a Superdex S75 column. The fresh protein eluted from the column was immediately reacted with a two-fold molar excess of the lanthanide-containing C2 tag, which incorporates a cysteine-reactive pyridin-2-yldisulfanyl moiety (Graham et al. 2011). Completion of the reaction was monitored through the appearance of a UV band at 345 nm corresponding to the released 2-mercaptopyridine group. The tagged protein fragment was used directly in SNARE complex assembly reactions.

SNARE complexes were assembled as previously described but with some modifications (Chen et al. 2002). Briefly, the SNARE motifs were mixed in the presence of 1 M NaCl to hinder oligomerization of the syntaxin-1 SNARE motif, rotating the mixture at room temperature overnight. Unlabeled fragments (including those containing a lanthanide tag) were added in 80% excess over the isotopically labeled fragment to facilitate full incorporation of the latter into SNARE complexes. The syntaxin-1 SNARE motif was added last to minimize formation of off-pathway complexes with the SNAP-25 SNARE motifs (Rizo and Sudhof 2012). Excess lanthanide-tag reagent and unassembled SNARE motifs were removed by five rounds of concentration-dilution with a 30 kDa molecular mass cutoff, the first three in 20 mM Tris (pH 7.4), 1 M NaCl, and the last two in 20 mM Tris (pH 7.4), 200 mM NaCl.

### NMR spectroscopy and PCS analysis

All NMR spectra were acquired at 25 °C on Varian INOVA spectrometers operating at 600 or 800 MHz equipped with cold probes. PCSs were measured from ^1^H-^13^C HMQC spectra (Tugarinov et al. 2004) of samples of SNARE complex that contained one of the SNARE motifs ^2^H,^13^CH_3_-ILMV-labeled and another SNARE motif with a lanthanide tag. Samples contained 20–30 µM SNARE complex dissolved in 20 mM Tris (pH 7.4), 200 mM NaCl and 5% D_2_O. Total acquisition times for the ^1^H-^13^C HMQC spectra ranged from 4 to 40 h. The spectra were processed with NMRPipe (Delaglio et al. 1995) and analyzed with NMRView (Johnson and Blevins 1994).

PCSs were calculated from the differences in the chemical shifts observed before and after removal of the lanthanide tag by reduction with 1 mM TCEP, which for the SNARE complex is equivalent to using a control with a diamagnetic tag (Brewer et al. 2015). The precision of the PCSs is estimated to be 0.008 ppm for PCSs measured on ^1^H chemical shifts and 0.05 ppm for those measured on ^13^C chemical shifts, although these are conservative limits based on the reproducibility observed in multiple measurements performed on analogous samples. Numbat (Schmitz et al. 2008) was used to derive Δχ tensors for given sets of PCSs. Assignment of ^1^H-^13^C HMQC cross-peaks to SNARE complex methyl groups was accomplished using an iterative procedure described in the “Results” section. We note that the placement of Trp residues at the N- or C-terminus of the SNAP-25 SNARE motifs to facilitate detection by UV spectroscopy (Chen et al. 2002; Xu et al. 2013) can alter the chemical shifts of nearby nuclei at the corresponding terminus. Assignments have been deposited in the BioMagResBank with accession number 12,003.

## Results

### Labeling strategy

The four-helix bundle of the neuronal SNARE complex is formed by one SNARE motif from each synaptobrevin (Syb) and syntaxin-1 (Syx), and two SNARE motifs from SNAP-25 (referred to as SNN and SNC for the N- and C-terminal SNARE motifs, respectively). The complex is highly stable (is SDS resistant) and hence can be handled as a single protein, but constitutes a favorable case to test PCS-based approaches for methyl resonance assignments because each SNARE motif can be isotopically labeled separately to simplify the NMR spectra. Conversely, some properties of the SNARE complex hinder assignment of methyl groups in addition to its limited solubility. Thus, the abundance of aliphatic residues and paucity of aromatic side chains leads to poor spectral dispersion, the resonances are broader than expected for a 32 kDa species because of the elongated shape of the complex (ca. 115 Å), and resonances from nuclei near a polar layer formed by three buried glutamines (Q226 from syntaxin-1, Q53 from SNN and Q174 from SNC) and one buried arginine from synaptobrevin (R56) (Sutton et al. 1998) exhibit additional broadening due to chemical exchange (Chen et al. 2002). Moreover, in our previous attempts to place lanthanide-loaded tags on the SNARE complex, we observed massive precipitation for several of the chelators tested and we were able to obtain soluble samples only when we used 1,4,7,10 tetraazacyclododecane-tetraacetic acid (DOTA)-based tags that have very high affinity for lanthanides and very slow off rates (Brewer et al. 2015).

The DOTA-based tag called C2 (no relation to the term C_2_ domain) was particularly useful and yielded large PCSs that could be fit to unique anisotropic magnetic susceptibility tensors (Δχ tensors) when loaded with Dy^3+^ and placed in four different positions of the N-terminal half of the SNARE complex (i.e. N-terminal to the polar layer). However, no interpretable PCSs were observed for the Dy^3+^-loaded C2 tag placed at five different positions within the C-terminal half of the complex. Four of these positions yielded strong broadening and only small PCSs, whereas for one position we observed large PCSs that could not be fit to a unique Δχ tensor (Brewer et al. 2015). This behavior likely arises because of motions in the C-terminal half of the SNARE complex that also underlie faster amide H/D exchange rates compared to those observed for the N-terminal half (Chen et al. 2002). Hence, our strategy to assign methyl resonances of the SNARE complex using PCSs necessarily relied on placing lanthanide-loaded C2 tags on positions in the N-terminal half of the complex even though some methyl groups at the very C-terminus of the complex are at distances of up to 60 Å from the tags. At the outset, this issue was not a problem because the large Δχ tensors associated with the Dy^3+^-loaded C2 tag yielded readily measurable PCSs (up to 0.04 ppm) at these distances.

**Fig. 1:**
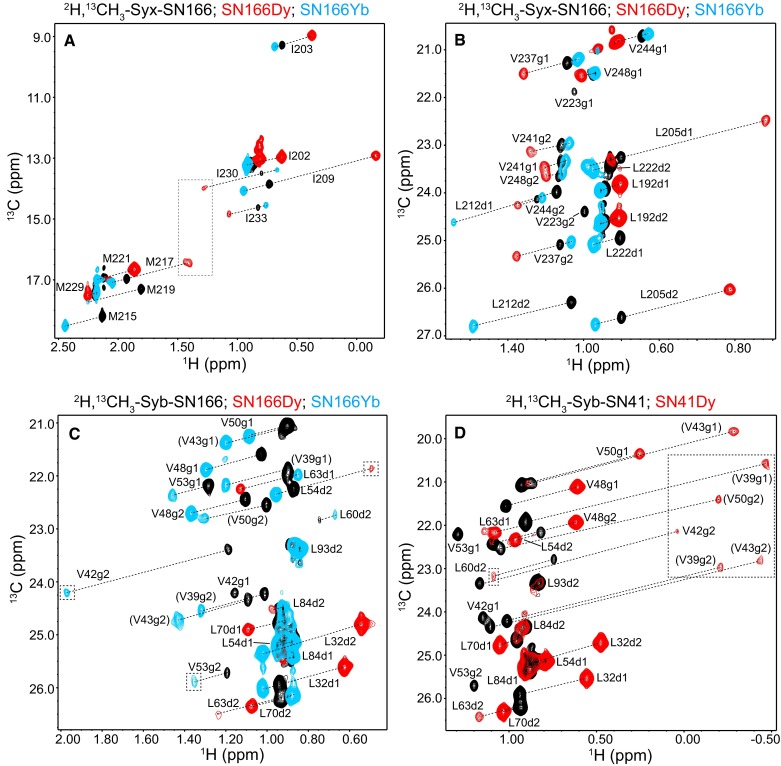
^1^H-^13^C HMQC spectra illustrating PCSs induced by lanthanide tags on methyl cross-peaks of the SNARE complex. **a–d** ^1^H-^13^C HMQC spectra of SNARE complexes containing ^2^H,^13^CH_3_-labeled syntaxin-1 (**a, b**) or synaptobrevin (**c, d**) SNARE motif that were untagged (*black* c*ontours*) or tagged at residue 166 or 41 of SNAP-25 with Dy^3+^-loaded C2 or Yb^3^-loaded C2 as indicated with the *color codes*. Regions of the spectra that were plotted at lower contour levels to allow visualization of weak cross-peaks are indicated by *dashed boxes*. The PCSs caused on selected cross-peaks are illustrated by *dashed lines* and the corresponding cross-peak assignments are indicated. *Parentheses* indicate tentative assignments

Based on all these considerations, our approach to assign the Ile-δ1, Leu, Met and Val methyl groups of the SNARE complex involved the acquisition of ^1^H-^13^C HMQC spectra on samples where only one of the SNARE motifs was perdeuterated and ^13^CH_3_-labeled at these methyl groups (^2^H,^13^CH_3_-ILMV-labeled), while the other three SNARE motifs where unlabeled. To measure PCSs, a Dy^3+^-loaded C2 tag was attached to single cysteine side chains placed by site-directed mutagenesis in one of three positions distributed within the N-terminal half of the SNARE complex: D41C or D166C of SNAP-25, or D214C of syntaxin-1. We will refer to SNARE complexes tagged at these different positions as SN41Dy, SN166Dy and Syx214Dy. We also prepared some samples where Yb^3+^-loaded C2 was placed on complexes containing the D166C mutation in SNAP-25 (referred to as SN166Yb). The samples used are summarized in Table 1 and Fig. 1 illustrates some of the PCSs observed with a diverse combination of ^2^H,^13^CH_3_-ILMV-labeled SNARE motifs, lanthanides and tag positions. PCS measurements were performed on samples where both methyl groups of Leu and Val residues were 50% ^13^CH_3_ labeled (Gardner and Kay 1998), but ^1^H-^13^C HMQC spectra of four SNARE complex samples where the Leu and Val methyl groups of one individual SNARE motif were ^13^CH_3_-labeled stereospecifically (Gans et al. 2010) were also acquired to help distinguish cross-peaks from pro-R and pro-S methyl groups.

**Table 1:** Summary of samples used for assignment of Ile-δ1, Leu, Met and Val methyl groups of the SNARE four-helix bundle

^2^H,^13^CH_3_-ILMV-labeled SNARE motif	Cysteine mutant	Lanthanide
Syx	SNAP-25 D41C	Dy^3+^
Syx	SNAP-25 D166C	Dy^3+^
Syx	SNAP-25 D166C	Yb^3+^
Syb	SNAP-25 D41C	Dy^3+^
Syb	SNAP-25 D166C	Dy^3+^
Syb	SNAP-25 D166C	Yb^3+^
SNN	SNAP-25 D166C	Dy^3+^
SNN	SNAP-25 D166C	Yb^3+^
SNN	Syntaxin-1 D214C	Dy^3+^
SNC	SNAP-25 D41C	Dy^3+^
SNC	Syntaxin-1 D214C	Dy^3+^

### PCS analysis

We had previously measured PCSs induced in backbone amide resonances of the SN41Dy, SN166Dy and Syx214Dy complexes for our studies of the synaptotagmin-1-SNARE complex structure, which were performed in the presence of 125 mM KSCN to prevent aggregation (Rizo and Xu 2015). Since we anticipate that standard salts such as NaCl, rather than the chaotropic KSCN, will be used for most structural studies, we obtained assignments of the Ile-δ1, Leu, Met and Val methyl groups of the SNARE complex in a buffer that contained 200 mM NaCl. This relatively high salt concentration was used to prevent aggregation, as SNARE complexes tagged with lanthanide-loaded C2 exhibit increased tendency to aggregate, but note that the amide chemical shifts of the SNARE are largely unaffected by changes in NaCl concentration from 100 to 300 mM (Chen et al. 2002). We attempted to use the Δχ tensors derived from the PCSs measured on backbone amide groups of the SN41Dy, SN166Dy and Syx214Dy complexes in the KSCN buffer (Brewer et al. 2015) to predict PCSs for methyl groups based on a high-resolution structure of the SNARE complex (Ernst and Brunger 2003), and then examined whether the predicted PCSs could be matched with those measured for methyl cross-peaks in ^1^H-^13^C HMQC spectra of these complexes. However, it became clear from this analysis that these Δχ tensors cannot be used to reliably assign many of the cross-peaks because the presence of KSCN leads to Δχ tensors that are substantially different from those existing under the conditions of our experiments. Indeed, comparison of ^1^H-^13^C HMQC spectra acquired in the 200 mM NaCl buffer and the 125 mM KSCN buffer showed that the PCSs induced in the SN166Dy complex were considerably different under the two conditions (Supplementary Fig. 1).

To obtain methyl group assignments, we first used the PCSs measured in SN41Dy, SN166Dy and Syx214Dy complexes (Table 1) and we followed an iterative procedure that started with the unambiguous or tentative assignment of some cross-peaks based on these PCSs and the high-resolution structure of the SNARE complex (Ernst and Brunger 2003). Most of these initial assignments corresponded to methyl groups from methionine or isoleucine groups, as there are only a few of these residues in each SNARE motif, and to some leucine and valine methyl groups that are far from the lanthanide tags. The latter assignments were made possible by the observation that the PCSs at the N-termini of the SNARE motifs were of opposite sign to those observed at the C-termini, and that their absolute values correlate with the distance to the lanthanide. For instance, the cross-peaks from the eight methyl groups of V237, V241, V244 and V248 at the C-terminal region of syntaxin-1 could be tentatively assigned based on their positive PCSs within the SN166Dy complex (Fig. 1b), the relative sizes of their PCSs and the knowledge on whether each cross-peak corresponds to a pro-R or pro-S methyl group. This information allows assignment of the I230 and I233 cross-peaks from syntaxin-1, which exhibit positive PCSs, and of the I202, I203 and I209 cross-peaks, which exhibit negative PCSs because these residues are on the other end of the SNARE complex (Fig. 1a). Small negative PCSs also yielded assignments for the L192 cross-peaks (Fig. 1b). Analogous arguments were used to obtain initial assignments for methyl groups from the other SNARE motifs.

To reduce ambiguities associated with the shape and symmetry of a single Δχ tensor (leading to similar PCSs for two or more cross-peaks), it was critical to measure the PCSs produced by attachment of the Dy^3+^-loaded C2 tag at different sites. Thus, for each SNARE complex with a different ^2^H,^13^CH_3_-ILMV-labeled SNARE motif, we measured PCSs induced by the Dy^3+^-loaded C2 tag at two different positions (Table 1). In addition to resolving ambiguities, the availability of two datasets for each SNARE motif helped to check the self-consistency of the assignments. For instance, the initial assignments made for syntaxin-1 using the SN166Dy data were confirmed with the SN41Dy data.

The initial cross-peak assignments and the measured PCSs were used to derive initial, approximate Δχ tensors for each each lanthanide-tagged complex (SN41Dy, SN166Dy and Syx214Dy). For this purpose, PCSs measured for different SNARE motifs were pulled together to derive the tensors (e.g. the Syx214Dy Δχ tensor was calculated from PCSs measured for the two SNARE motifs of SNAP-25, SNN and SNC). The metal position was assumed to be the same regardless of which SNARE motif was isotopically labeled. These initial Δχ tensors were then used to predict PCSs for all methyl groups. Comparison of these predictions with the measured PCSs led to additional assignments that helped to refine the Δχ tensors, and the refined tensors yielded more accurate predictions that allowed additional assignments that were previously unclear. This procedure was repeated multiple times to obtain as many assignments as possible with the complexes labeled with the Dy^3+^-loaded C2 tag. It is noteworthy that Δχ tensors obtained initially were considerably different from the final ones, with much larger rhombic components. However, this observation arises because a limited set of PCSs can be fit with markedly different Δχ tensors. The robustness of the initial methyl assignments was verified at the outset by the fact that the associated PCSs were fully consistent with those predicted for the relevant methyl groups at the end of the iterative procedure.

Some ambiguities remained after thorough analysis of the PCSs measured for the SN41Dy, SN166Dy and Syx214Dy complexes, in part because Dy^3+^ caused broadening beyond detection of cross-peaks from methyl groups in its proximity (ca. 20 Å or less) such that PCSs could not be measured for these cross-peaks. To resolve these ambiguities, we measured PCSs in three complexes tagged with Yb^3+^-loaded C2 (SN166Yb), as the paramagnetic broadening caused by Yb^3+^ is much weaker than that caused by Dy^3+^ [(Otting 2010); see also Fig. 1a–c]. With the cross-peak assignments that were already available, we were able to immediately interpret many of the PCSs caused by the Yb^3+^-loaded C2 tag in SN166Yb samples and hence derive an accurate Δχ tensor that in turn helped us to resolve most of the ambiguities remaining in the assignments, together with the PCSs induced by the Dy^3+^-loaded C2 tag.

### Methyl resonance assignments of the SNARE complex

**Fig. 2:**
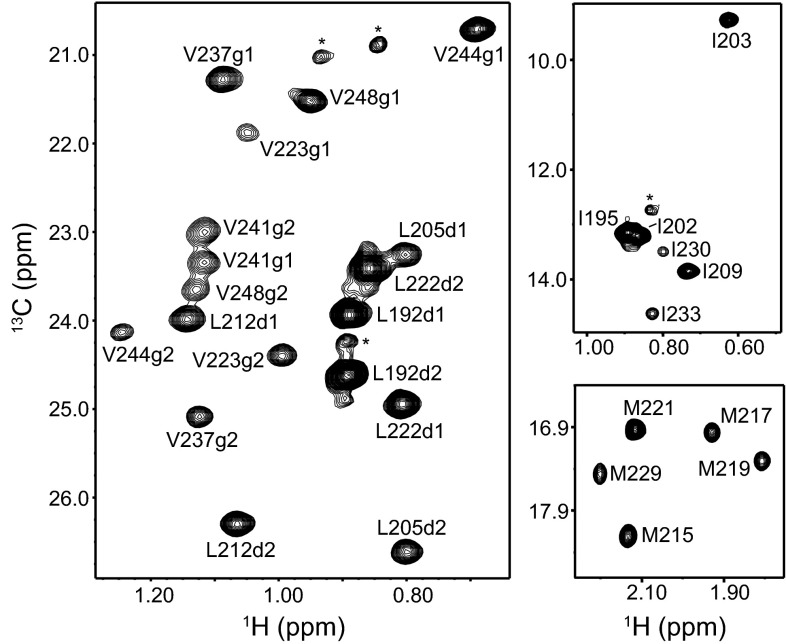
Methyl cross-peak assignments of the syntaxin-1 SNARE motif within the SNARE complex. Different expansions of a ^1^H-^13^C HMQC spectrum of a SNARE complex containing ^2^H,^13^CH_3_-labeled syntaxin-1 SNARE motif are shown. *Cross-peak* assignments are indicated. *Cross-peaks* corresponding to a small amount of unassembled syntaxin-1 SNARE motif are denoted by *asterisk*

**Fig. 3:**
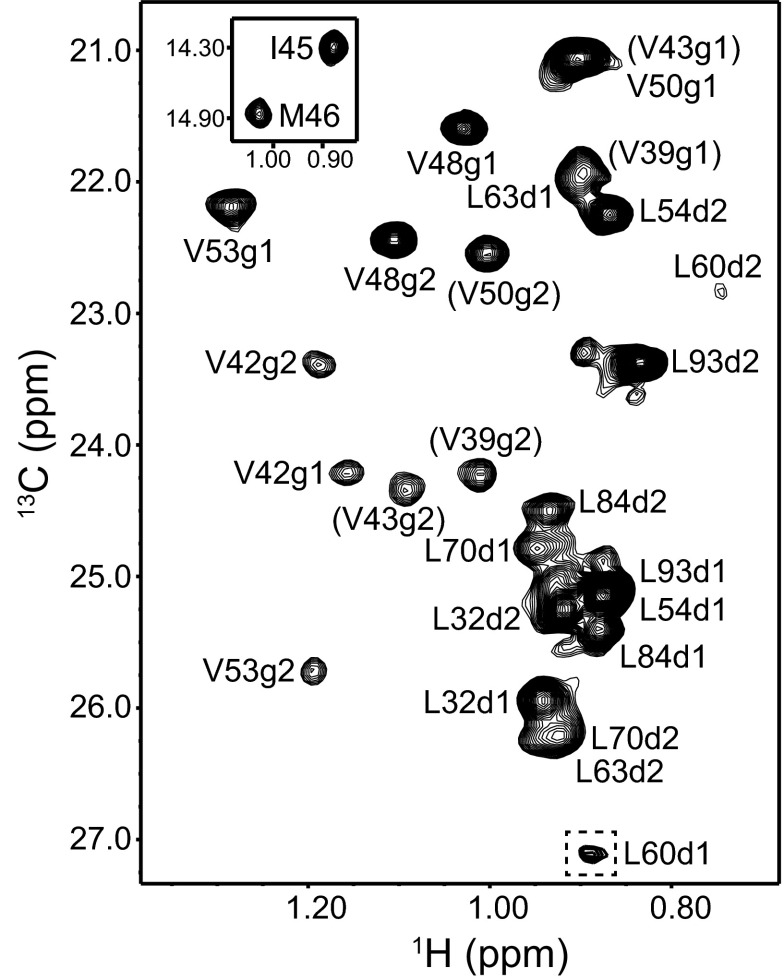
Methyl cross-peak assignments of the synaptobrevin SNARE motif within the SNARE complex. An expansion corresponding to the Leu-Val region of a ^1^H-^13^C HMQC spectrum of a SNARE complex containing ^2^H,^13^CH_3_-labeled synaptobrevin SNARE motif is shown. The *inset* shows an expansion of the region containing the two cross-peaks corresponding to Ile and Met (note that the M46 cross-peak appears strongly shifted upfield compared to the usual positions of Met methyl cross-peaks). Cross-peak assignments are indicated. *Parentheses* indicate tentative assignments

**Fig. 4:**
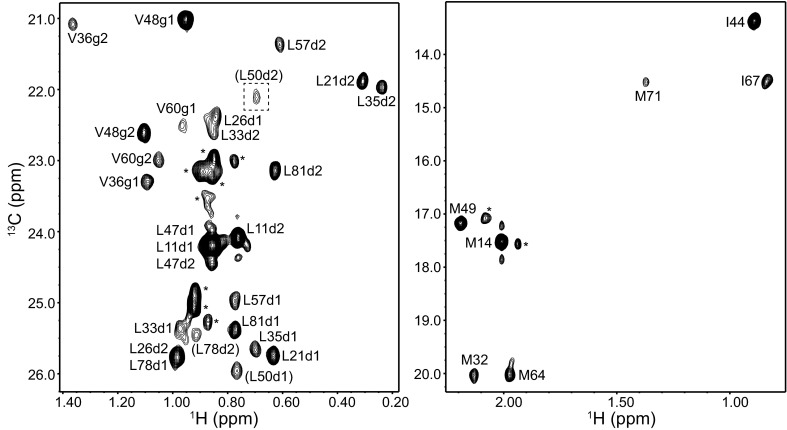
Methyl cross-peak assignments of the SNAP-25 N-terminal SNARE motif (SNN) within the SNARE complex. Different expansions of a ^1^H-^13^C HMQC spectrum of a SNARE complex containing ^2^H,^13^CH_3_-labeled SNN are shown. A region of the spectrum that was plotted at lower contour levels to allow visualization of a weak cross-peak is indicated by a *dashed box. Cross-peak* assignments are indicated. *Parentheses* indicate tentative assignments. Cross-peaks corresponding to a small amount of unassembled SNN are denoted by *asterisk*

**Fig. 5:**
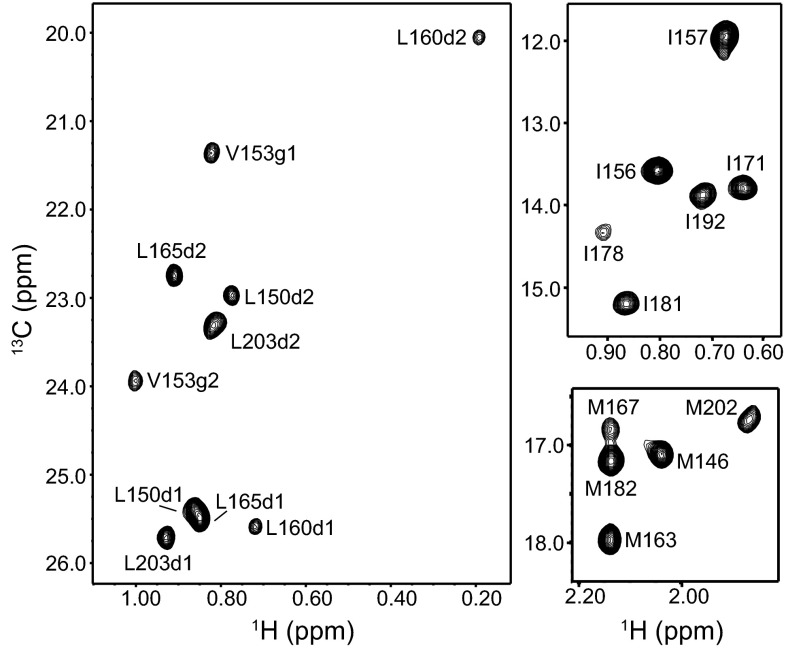
Methyl cross-peak assignments of the SNAP-25 C-terminal SNARE motif (SNC) within the SNARE complex. Different expansions of a ^1^H-^13^C HMQC spectrum of a SNARE complex containing ^2^H,^13^CH_3_-labeled SNC are shown. *Cross-peak* assignments are indicated

The procedure outlined above, with some nuances noted below, allowed us to obtain unambiguous assignments for 103 out of the 111 (93%) Ile-δ1, Leu, Met and Val methyl groups of the SNARE complex, and tentative assignments for the remaining eight methyl groups (Figs. 2, 3, 4, 5), using a total of 349 measured PCSs (Supplementary Table 1). These included 82, 108 and 90 PCSs for ^1^H and ^13^C methyl nuclei from the SN41Dy, SN166Dy and Syx214Dy complexes, respectively. For the SN166Yb complex, we measured only ^1^H PCSs because the shifts were generally smaller, and hence a considerable number of the PCSs observed for ^13^C nuclei were close to the uncertainty of the ^13^C chemical shift measurements. Moreover, because of the lower degree of broadening caused by Yb^3+^, PCSs for more cross-peaks (a total of 69) were observable for this complex and hence the ^13^C PCSs were less necessary to derive an accurate Δχ tensor.

Assignment of the methyl groups from the syntaxin-1 SNARE motif (Fig. 2) were obtained with PCSs induced in SN41Dy, SN166Dy and SN166Yb complexes. As explained above, many assignments were straightforward based only on the SN166Dy PCSs, and these assignments were confirmed with the SN41Dy PCSs. Assignments for the methyl groups that are close to the tags (those of Met 215, 217 and 219; Leu 212 and 222; and Val 223) were obtained from the PCSs observed for the SN166Yb complex (Fig. 1a, b). Note that the cross-peaks of Val 223 are very broad (Fig. 2) because this residue is close to the polar layer and that the corresponding cross-peaks in the SN166Yb complex are not observable at the level plotted in Fig. 1b, but these cross-peaks are nevertheless present and reveal PCSs consistent with those predicted with the final calculated Δχ tensor.

SN41Dy, SN166Dy and SN166Yb complexes were also used to assign the methyl groups of the synaptobrevin SNARE motif (Fig. 3). In this case, the methyl groups of the single methionine and isoleucine residues, and of most of the leucines, could readily be assigned from the SN41Dy and SN166Dy PCSs. The Leu 60 methyl groups are adjacent to the polar layer and, correspondingly, are very broad, but we could assign them based on the PCSs observed for the SN41Dy and SN166Yb complexes. Assignment of the methyl groups from the five valine residues of the synaptobrevin SNARE motif was hindered because all of them are in the N-terminal half of the SNARE complex and hence relatively close to the lanthanide tags. No cross-peaks for the valine methyl groups could be observed for the SN166Dy complex (Fig. 1c, red contours) even though the distances between some of them and the Dy^3+^ ion are in the 22–23 Å range, whereas cross-peaks were observed for most of the valine methyl groups for the SN41Dy complex (Fig. 1d, red contours), including for some methyl groups located 19–20 Å from the Dy^3+^ ion. These findings suggest that some motions of the tag in the SN166Dy complex may cause additional broadening due to chemical exchange and/or enhanced paramagnetic relaxation when the tag comes close to a given methyl group. We did observe cross-peaks for most valine methyl groups of the SN166Yb complex (Fig. 1c, cyan contours), which, together with the SN41Dy PCSs, yielded assignments for some of the valine methyl groups and tentative assignments for the others (indicated in parenthesis in Fig. 3). Although these tentative assignments are likely to be correct because they fit best with the final Δχ tensors derived from the remaining data, we cannot be 100% certain because the PCSs observed in SN41Dy and SN166Yb complexes for these methyl groups are all large and similar in size, and even slight inaccuracies in the structure of the SNARE complex or the tensors can cause large variations in the PCSs predicted for methyl groups that are so close to the lanthanides. We did not attempt to solve these ambiguities using PCSs measured with Syx214Dy or Syx214Yb complexes because residue 214 of syntaxin is very close to the corresponding methyl groups.

In the ^1^H-^13^C HMQC spectra of SNARE complexes that were ^2^H,^13^CH_3_-ILMV-labeled in the SNAP-25 N-terminal SNARE motif (SNN) we observed numerous cross-peaks corresponding to isolated SNN (marked with a asterisk in Fig. 4). Such cross-peaks arise from small amounts of SNN that did not assemble into the SNARE complex but yield strong cross-peaks because of their high flexibility. Cross-peaks from unassembled SNARE motif were also observed sometimes in the spectra of complexes with the other SNARE motifs ^2^H,^13^CH_3_-ILMV-labeled, particularly for the syntaxin-1 SNARE motif (Fig. 2), but they were readily distinguished from those of the SNARE complex because of their sharp nature and the lack of PCSs, as well as by comparison with spectra obtained for the isolated SNARE motifs. Most of the methyl group assignments for SNN within the SNARE complex (Fig. 4) were obtained from PCSs measured on SN166Dy and Syx214Dy complexes, and most of the remaining assignments were obtained from these PCSs in combination with those measured with the SN166Yb complex. Only three assignments are tentative because of resonance overlap and/or lack of observed PCSs due to resonance broadening. Finally, assignment of the methyl groups from the SNAP-25 C-terminal SNARE motif (SNC) (Fig. 5) did not present any particular problems. We note that the cross-peaks from Leu 50 of SNN and Ile 178 of SNC were broad, as expected from their proximity to the polar layer.

**Fig. 6:**
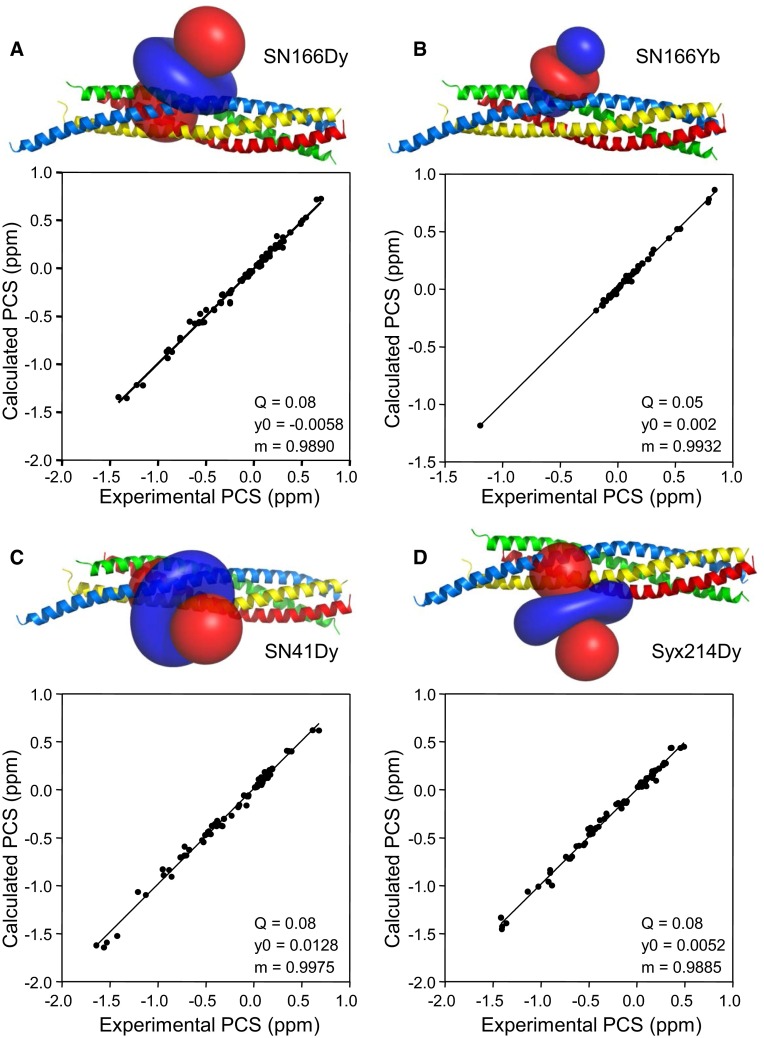
Anisotropic magnetic susceptibility tensors and correlations of observed versus predicted PCSs. **a–d** The *top* of each panel shows a *ribbon diagram* of the SNARE complex (syntaxin-1 in *yellow*, synaptobrevin in *red*, SNN in *blue* and SNC in *green*) (PDB code 1N7S) with isosurfaces representing regions with positive (*blue*) or negative (*red*) PCSs, contoured at ±0.8 ppm, with the Δχ tensors derived for SN166Dy (**a**), SN166Yb (**b**), SN41Dy (**c**) and Syx214Dy (**d**). The *bottom* of each panel shows the correlation between experimental PCSs measured for each complex and those calculated with the corresponding Δχ tensor. Q factors (*Q*), slopes (*m*) and y axis intercepts (*y0*) are indicated. Q factors were calculated as the root mean square deviation between measured and calculated PCSs divided by the root mean square of the measured PCSs

Table 2 lists the parameters that describe the final Δχ tensors derived for the SN166Dy, SN166Yb, SN41Dy and Syx214Dy complexes. Figure 6 illustrates the shapes of the tensors and the correlations between measured and predicted PCSs for each tensor. All Q factors (Q) are excellent, the slopes (m) are close to 1 and the y0 intercepts are close to 0. For each complex, the derived metal position is close to the side chain that was tagged, within a distance that is fully compatible with the covalent structure of the complex. Correspondingly, the SN166Yb tensor has a similar center and orientation as the SN166Dy tensor, but is smaller in size and opposite in sign (Table 2; Fig. 6a, b), as expected from the paramagnetic properties of Dy^3+^ and Yb^3+^ in complex with the C2 tag (Graham et al. 2011). The opposite sign underlies the opposite directions of the PCSs observed for the SN166Dy and SN166Yb complexes (Fig. 1a–c). All these observations show the overall consistency of the data and support the validity of the assignments. However, the quality of the tensors and the fits between measured and predicted PCSs need to be interpreted with caution, as it is likely that the uncertainty in the tensor parameters is larger than the estimated errors described in Table 2 because of potential structural inaccuracies and because the tensor parameters are strongly influenced by the larger PCSs, among other reasons. For instance, removal of the PCSs measured for a given methyl in a particular complex and recalculation of the corresponding tensor generally leads to more marked alterations of the tensor parameters and worsening of the predicted PCSs for the given methyl if the PCSs are large than if they are small. Note also that the Δχ tensors obtained for SN166Dy and SN41Dy are considerably different from those obtained previously for these same complexes in buffer containing KSCN (Brewer et al. 2015), illustrating how the solution conditions can alter the resulting Δχ tensors. We speculate that these differences arise because the SNARE complex is highly charged and some of its exposed side chains may interact differently with these salts, as suggested by the observation that KSCN is more efficient in hindering precipitation of the SNARE complex (Brewer et al. 2015).

**Table 2:** Magnetic susceptibility tensors derived during the assignment of the SNARE complex I, L, M and V methyl groups

Tensor	Δχ_ax_^a^	Δχ_rh_^a^	X^b^	Y^b^	Z^b^	Tensor axis	Coordinates of tensor axes^c^		
SN166Dy	−26.9	−5.4	10.6	58.5	10.4	x	−0.997	0.042	0.064
	(±1.1)	(±1.1)	(±0.7)	(±0.6)	(±0.5)	y	0.076	0.491	0.868
						z	0.004	0.870	−0.493
SN166Yb	7.9	1.9	11.4	59.4	10.2	x	−0.948	0.276	0.16
	(±0.4)	(±0.4)	(±0.3)	(±0.4)	(±0.5)	y	0.256	0.359	0.898
						z	0.190	0.891	−0.411
SN41Dy	−24.1	−2.3	22.3	53.6	29.8	x	−0.512	0.421	0.749
	(±0.6)	(±0.9)	(±0.3)	(±0.7)	(±0.5)	y	0.142	−0.818	0.557
						z	0.848	0.391	0.359
Syx214Dy	−21.2	−5.1	21.5	35.0	26.5	x	−0.571	−0.42	−0.706
	(±0.6)	(±1.4)	(±0.5)	(±0.7)	(±0.8)	y	−0.694	−0.214	0.688
						z	−0.439	0.882	−0.169

The tensors are listed in their unique tensor representation (UTR) as derived by simultaneously fitting all the PCSs measured for a given lanthanide at a given site (Table 1, Supplementary Table 1) using Numbat, assuming a common metal position for samples istopically labeled at different SNARE motifs but containing the same lanthanide at the same site. The errors estimated for Δχ_ax_ and Δχ_rh_, as well as for the tensor center coordinates, are indicated in parenthesis below their values. The errors correspond to the standard deviations yielded by Numbat using a Monte Carlo error analysis with 100 samples and random omission of 20% of the PCSs for each Monte Carlo sample

^a^Tensor parameters in units of 10^−32^ m^3^

^b^Coordinates of tensor center in the coordinate system used in the high-resolution structure of the SNARE complex (Ernst and Brunger 2003) (PDB accession code 1N7S)

^c^The tensor axes are given as unit vectors with respect to the origin (0, 0, 0)

## Discussion

The machinery that controls Ca^2+^-triggered neurotransmitter release has been extensively characterized but fundamental questions remain about how this machinery triggers Ca^2+^-dependent membrane fusion, in part because structural studies have mostly used soluble proteins. A major leap forward to reach a clear understanding of the mechanism of release will necessitate analysis of protein complexes in their native membrane environment, ideally between two lipid bilayers. Application of solution NMR methods using methyl TROSY spectra in combination with small nanodisc bilayers (Hagn et al. 2013) provides a promising avenue for this purpose, but a key hurdle is the need to obtain resonance assignments of the methyl groups of central components that have limited solubility. The study presented here yields the assignments of the Ile, Leu, Met and Val methyl groups of the SNARE complex and provides an illustration of the usefulness of lanthanide-induced PCSs to assign methyl groups of proteins or protein complexes of known structure.

The SNARE complex constitutes a favorable target to apply this approach because of its relatively small size and the possibility of labeling each helix individually, but also presents some difficulties because of poor chemical shift dispersion and strong broadening in some cross-peaks. Some of the broadening arises through anisotropic tumbling from the elongated shape of the SNARE complex and because, for each sample with a given SNARE motif ^2^H,^13^CH_3_-ILMV-labeled, the other three helices were not perdeuterated. Hence, there is still a relatively high proton density around methyl groups that pack against the other helices, limiting the benefits of the methyl TROSY approach for these methyl groups. Nevertheless, the resulting broadening, and the even stronger broadening observed for cross-peaks from methyl groups located near the polar layer due to chemical exchange, did not prevent the assignment of methyl groups using the PCS-based approach at the relatively low SNARE complex concentrations used (20–30 µM). Similarly, we do not expect that the resonance broadening characteristic of much larger protein complexes (e.g. in the hundreds of kDa range) should prevent application of this approach.

Our study also benefited from two additional factors. First, a high-resolution structure of the SNARE complex was available, which is required to predict accurate PCSs. The high quality of the structure is particularly crucial for methyl groups close to the lanthanide tags, as small shifts in nuclei positions can yield large differences in the calculated PCSs. Second, some methyl groups of the SNARE complex could be assigned readily, particularly those of Met and Ile residues, thus allowing the calculation of initial approximate tensors that were used as starting points for the manual iterative procedure that we followed to assign the SNARE complex methyl groups. Clearly, computational approaches can greatly facilitate the PCS-based assignment process (John et al. 2007; Skinner et al. 2013) and, in challenging cases such as large proteins with extensive overlap, a few initial unambiguous assignments can be obtained through mutagenesis and/or with the help of chemical shift prediction programs (Wishart 2011).

A major problem that can be expected in the application of the PCS-based approach to assign methyl groups of large proteins is spectral overlap, but it is important to note that, even if only partial methyl group assignments are obtained, such assignments still provide very powerful tools for structural analyses. Moreover, lanthanide-induced PCSs can be used to increase the cross-peak dispersion in the ^1^H-^13^C HMQC spectra (e.g. Fig. 1). Hence, a potential strategy to alleviate the spectral overlap is to use ^2^H,^13^CH_3_-labeled protein with a given lanthanide tag (e.g. Yb^3+^-loaded C2) at a particular residue (e.g. residue X) and assign the better-resolved ^1^H-^13^C HMQC spectrum of such protein using PCSs caused by lanthanide tags at other residues. The ^2^H,^13^CH_3_-labeled protein with the Yb^3+^-loaded C2 tag at residue X can then be used as a subunit for structural studies of complexes with target proteins. This strategy requires the use of orthogonal reactions for labeling the same protein with two lanthanide tags, which should be feasible due to the continued development of methodology to introduce non-canonical amino acid residues into proteins (O’Donoghue et al. 2013). This methodology also facilitates the introduction of lanthanide tags in proteins that have multiple native cysteines and hence are difficult to tag selectively at a single cysteine residue.

An important aspect of the PCS-based approach for methyl resonance assignment that emerged in our study is the choice of tag location. A minor issue in this respect is the fact that introduction of cysteine mutations can alter the chemical shifts of nearby methyl groups. For instance, the D214C mutation in syntaxin-1 caused substantial shifts in the nearby L35 methyl groups of SNAP-25. However, the perturbations caused by the mutations used in our study did not really cause difficulties, as they were very limited, and in fact helped to confirm these assignments. A more important problem associated with the choice of tag location is the potential for tag mobility, which can lead to complete absence of PCSs (because they are averaged to zero) or averaging of the Δχ tensor that can complicate the interpretation of the measured PCSs (Shishmarev and Otting 2013). In our previous study, the Dy^3+^-loaded C2 tag led to the observation of PCSs that could be fit to a unique Δχ tensor when placed at four out of ten sites in the SNARE complex (Brewer et al. 2015). Note also that the buffer conditions can alter the mobility and/or tag position, as the presence of 200 mM NaCl or 125 mM KSCN in the buffer can yield different PCSs (Supplementary Fig. 1) and considerably different Δχ tensors (Table 2 and Brewer et al. 2015). Tag mobility can be limited through the design of the tag, for instance by increasing its bulk (Graham et al. 2011), or by anchoring it to two cysteines, which can in some cases offer predictable Δχ tensors without the need for initial assignments (Hass and Ubbink 2014; Lee et al. 2016). It is likely that computational methods including molecular dynamics simulations can help identifying appropriate positions where lanthanide tags can be suitably placed in a rigid environment. These considerations will be important to undertake the assignment of methyl groups of challenging targets, including crucial components of the release machinery such as Munc18-1 and Munc13-1.

## Electronic supplementary material

Below is the link to the electronic supplementary material.

Supplementary material 1 (DOCX 45 KB)

Supplementary material 2 (PDF 473 KB)
