# Supplementary material for: Sequence-specific assignment of methyl groups from the neuronal SNARE complex using lanthanide-induced pseudocontact shifts
Source: J Biomol NMR. 2016 Dec 17;66(4):281–93. doi: 10.1007/s10858-016-0078-1 (PMC5216067; doi:10.1007/s10858-016-0078-1)
Supplement: Supplementary file 1 — Supplementary material 1 (DOCX 45 KB) [file 10858_2016_78_MOESM1_ESM.docx]

**Supplementary Table 1.** PCSs measured in SNARE complexes with distinct lanthanide tags^*^.

Lanthanide SNARE

Tag motif residue methyl PCS (ppm)

| SN166Dy | SNN | L | 11 | CD1 | -0.023 |
| --- | --- | --- | --- | --- | --- |
| SN166Dy | SNN | L | 11 | CD2 | -0.017 |
| SN166Dy | SNN | L | 11 | HD1* | -0.044 |
| SN166Dy | SNN | L | 11 | HD2* | -0.032 |
| SN166Dy | SNN | M | 14 | CE | -0.046 |
| SN166Dy | SNN | M | 14 | HE* | -0.056 |
| SN166Dy | SNN | L | 21 | CD1 | -0.113 |
| SN166Dy | SNN | L | 21 | CD2 | -0.139 |
| SN166Dy | SNN | L | 21 | HD1* | -0.113 |
| SN166Dy | SNN | L | 21 | HD2* | -0.123 |
| SN166Dy | SNN | L | 26 | CD1 | -0.075 |
| SN166Dy | SNN | L | 26 | CD2 | -0.046 |
| SN166Dy | SNN | L | 26 | HD1* | -0.059 |
| SN166Dy | SNN | L | 26 | HD2* | -0.036 |
| SN166Dy | SNN | M | 32 | CE | -0.412 |
| SN166Dy | SNN | M | 32 | HE* | -0.416 |
| SN166Dy | SNN | L | 35 | CD1 | -0.535 |
| SN166Dy | SNN | L | 35 | CD2 | -0.518 |
| SN166Dy | SNN | L | 35 | HD1* | -0.555 |
| SN166Dy | SNN | L | 35 | HD2* | -0.531 |
| SN166Dy | SNN | V | 36 | CG1 | -1.155 |
| SN166Dy | SNN | V | 36 | CG2 | -0.897 |
| SN166Dy | SNN | V | 36 | HG1* | -1.220 |
| SN166Dy | SNN | V | 36 | HG2* | -0.852 |
| SN166Dy | SNN | L | 57 | CD1 | 0.654 |
| SN166Dy | SNN | L | 57 | CD2 | 0.543 |
| SN166Dy | SNN | L | 57 | HD1* | 0.699 |
| SN166Dy | SNN | L | 57 | HD2* | 0.508 |
| SN166Dy | SNN | V | 60 | CG1 | 0.240 |
| SN166Dy | SNN | V | 60 | CG2 | 0.379 |
| SN166Dy | SNN | V | 60 | HG1* | 0.303 |
| SN166Dy | SNN | V | 60 | HG2* | 0.381 |
| SN166Dy | SNN | M | 64 | CE | 0.175 |
| SN166Dy | SNN | M | 64 | HE* | 0.219 |
| SN166Dy | SNN | I | 67 | CD1 | 0.165 |
| SN166Dy | SNN | I | 67 | HD1* | 0.155 |
| SN166Dy | SNN | M | 71 | CE | 0.086 |
| SN166Dy | SNN | M | 71 | HE* | 0.084 |
| SN166Dy | SNN | L | 78 | CD1 | 0.072 |
| SN166Dy | SNN | L | 78 | HD1* | 0.059 |
| SN166Dy | SNN | L | 81 | CD1 | 0.034 |
| SN166Dy | SNN | L | 81 | CD2 | 0.028 |
| SN166Dy | SNN | L | 81 | HD1* | 0.034 |
| SN166Dy | SNN | L | 81 | HD2* | 0.029 |
| SN166Dy | synaptobrevin | L | 32 | CD1 | -0.332 |
| SN166Dy | synaptobrevin | L | 32 | CD2 | -0.351 |
| SN166Dy | synaptobrevin | L | 32 | HD1* | -0.324 |
| SN166Dy | synaptobrevin | L | 32 | HD2* | -0.342 |
| SN166Dy | synaptobrevin | L | 54 | CD2 | -0.341 |
| SN166Dy | synaptobrevin | L | 54 | HD2* | -0.343 |
| SN166Dy | synaptobrevin | L | 63 | CD1 | 0.303 |
| SN166Dy | synaptobrevin | L | 63 | CD2 | 0.279 |
| SN166Dy | synaptobrevin | L | 63 | HD1* | 0.229 |
| SN166Dy | synaptobrevin | L | 63 | HD2* | 0.312 |
| SN166Dy | synaptobrevin | L | 70 | CD1 | 0.166 |
| SN166Dy | synaptobrevin | L | 70 | CD2 | 0.153 |
| SN166Dy | synaptobrevin | L | 70 | HD1* | 0.140 |
| SN166Dy | synaptobrevin | L | 70 | HD2* | 0.146 |
| SN166Dy | synaptobrevin | L | 84 | CD1 | 0.045 |
| SN166Dy | synaptobrevin | L | 84 | CD2 | 0.049 |
| SN166Dy | synaptobrevin | L | 84 | HD1* | 0.046 |
| SN166Dy | synaptobrevin | L | 84 | HD2* | 0.043 |
| SN166Dy | syntaxin-1 | L | 192 | CD1 | -0.077 |
| SN166Dy | syntaxin-1 | L | 192 | CD2 | -0.088 |
| SN166Dy | syntaxin-1 | L | 192 | HD1* | -0.078 |
| SN166Dy | syntaxin-1 | L | 192 | HD2* | -0.075 |
| SN166Dy | syntaxin-1 | I | 195 | CD1 | -0.085 |
| SN166Dy | syntaxin-1 | I | 195 | HD1* | -0.074 |
| SN166Dy | syntaxin-1 | I | 202 | CD1 | -0.243 |
| SN166Dy | syntaxin-1 | I | 202 | HD1* | -0.232 |
| SN166Dy | syntaxin-1 | I | 203 | CD1 | -0.260 |
| SN166Dy | syntaxin-1 | I | 203 | HD1* | -0.245 |
| SN166Dy | syntaxin-1 | L | 205 | CD1 | -0.766 |
| SN166Dy | syntaxin-1 | L | 205 | CD2 | -0.577 |
| SN166Dy | syntaxin-1 | L | 205 | HD1* | -0.770 |
| SN166Dy | syntaxin-1 | L | 205 | HD2* | -0.571 |
| SN166Dy | syntaxin-1 | I | 209 | CD1 | -0.909 |
| SN166Dy | syntaxin-1 | I | 209 | HD1* | -0.888 |
| SN166Dy | syntaxin-1 | M | 215 | CE | -1.411 |
| SN166Dy | syntaxin-1 | M | 215 | HE* | -1.323 |
| SN166Dy | syntaxin-1 | M | 217 | CE | -0.560 |
| SN166Dy | syntaxin-1 | M | 217 | HE* | -0.499 |
| SN166Dy | syntaxin-1 | M | 221 | CE | -0.248 |
| SN166Dy | syntaxin-1 | M | 221 | HE* | -0.248 |
| SN166Dy | syntaxin-1 | L | 222 | CD1 | -0.669 |
| SN166Dy | syntaxin-1 | L | 222 | HD1* | -0.615 |
| SN166Dy | syntaxin-1 | M | 229 | CE | 0.076 |
| SN166Dy | syntaxin-1 | M | 229 | HE* | 0.059 |
| SN166Dy | syntaxin-1 | I | 230 | CD1 | 0.489 |
| SN166Dy | syntaxin-1 | I | 230 | HD1* | 0.490 |
| SN166Dy | syntaxin-1 | I | 233 | CD1 | 0.227 |
| SN166Dy | syntaxin-1 | I | 233 | HD1* | 0.254 |
| SN166Dy | syntaxin-1 | V | 237 | CG1 | 0.239 |
| SN166Dy | syntaxin-1 | V | 237 | CG2 | 0.259 |
| SN166Dy | syntaxin-1 | V | 237 | HG1* | 0.232 |
| SN166Dy | syntaxin-1 | V | 237 | HG2* | 0.231 |
| SN166Dy | syntaxin-1 | V | 241 | CG1 | 0.132 |
| SN166Dy | syntaxin-1 | V | 241 | CG2 | 0.132 |
| SN166Dy | syntaxin-1 | V | 241 | HG1* | 0.086 |
| SN166Dy | syntaxin-1 | V | 241 | HG2* | 0.163 |
| SN166Dy | syntaxin-1 | V | 244 | CG1 | 0.108 |
| SN166Dy | syntaxin-1 | V | 244 | CG2 | 0.123 |
| SN166Dy | syntaxin-1 | V | 244 | HG1* | 0.119 |
| SN166Dy | syntaxin-1 | V | 244 | HG2* | 0.104 |
| SN166Dy | syntaxin-1 | V | 248 | CG1 | 0.072 |
| SN166Dy | syntaxin-1 | V | 248 | CG2 | 0.053 |
| SN166Dy | syntaxin-1 | V | 248 | HG1* | 0.058 |
| SN166Dy | syntaxin-1 | V | 248 | HG2* | 0.069 |
| SN166Yb | SNN | L | 11 | HD1* | 0.006 |
| SN166Yb | SNN | L | 11 | HD2* | 0.011 |
| SN166Yb | SNN | M | 14 | HE* | 0.008 |
| SN166Yb | SNN | L | 21 | HD1* | 0.021 |
| SN166Yb | SNN | L | 21 | HD2* | 0.026 |
| SN166Yb | SNN | M | 32 | HE* | 0.094 |
| SN166Yb | SNN | L | 35 | HD1* | 0.131 |
| SN166Yb | SNN | L | 35 | HD2* | 0.126 |
| SN166Yb | SNN | V | 36 | HG1* | 0.295 |
| SN166Yb | SNN | V | 36 | HG2* | 0.212 |
| SN166Yb | SNN | V | 48 | HG1* | -1.194 |
| SN166Yb | SNN | M | 49 | HE* | -0.124 |
| SN166Yb | SNN | L | 57 | HD1* | -0.188 |
| SN166Yb | SNN | L | 57 | HD2* | -0.134 |
| SN166Yb | SNN | V | 60 | HG2* | -0.102 |
| SN166Yb | SNN | M | 64 | HE* | -0.060 |
| SN166Yb | SNN | I | 67 | HD1* | -0.040 |
| SN166Yb | SNN | M | 71 | HE* | -0.023 |
| SN166Yb | SNN | L | 78 | HD2* | -0.010 |
| SN166Yb | SNN | L | 81 | HD1* | -0.014 |
| SN166Yb | SNN | L | 81 | HD2* | -0.012 |
| SN166Yb | synaptobrevin | L | 32 | HD1* | 0.080 |
| SN166Yb | synaptobrevin | L | 32 | HD2* | 0.102 |
| SN166Yb | synaptobrevin | V | 42 | HG1* | 0.842 |
| SN166Yb | synaptobrevin | V | 42 | HG2* | 0.786 |
| SN166Yb | synaptobrevin | I | 45 | HD1* | 0.792 |
| SN166Yb | synaptobrevin | V | 48 | HG1* | 0.268 |
| SN166Yb | synaptobrevin | V | 48 | HG2* | 0.263 |
| SN166Yb | synaptobrevin | V | 50 | HG1* | 0.184 |
| SN166Yb | synaptobrevin | V | 53 | HG1* | 0.175 |
| SN166Yb | synaptobrevin | V | 53 | HG2* | 0.158 |
| SN166Yb | synaptobrevin | L | 54 | HD1* | 0.073 |
| SN166Yb | synaptobrevin | L | 54 | HD2* | 0.088 |
| SN166Yb | synaptobrevin | L | 60 | HD1* | -0.073 |
| SN166Yb | synaptobrevin | L | 60 | HD2* | -0.076 |
| SN166Yb | synaptobrevin | L | 63 | HD1* | -0.052 |
| SN166Yb | synaptobrevin | L | 63 | HD2* | -0.066 |
| SN166Yb | synaptobrevin | L | 70 | HD1* | -0.032 |
| SN166Yb | synaptobrevin | L | 70 | HD2* | -0.047 |
| SN166Yb | synaptobrevin | L | 84 | HD1* | -0.009 |
| SN166Yb | synaptobrevin | L | 84 | HD2* | -0.012 |
| SN166Yb | syntaxin-1 | L | 192 | HD1* | 0.018 |
| SN166Yb | syntaxin-1 | L | 192 | HD2* | 0.016 |
| SN166Yb | syntaxin-1 | I | 195 | HD1* | 0.021 |
| SN166Yb | syntaxin-1 | I | 203 | HD1* | 0.060 |
| SN166Yb | syntaxin-1 | L | 205 | HD1* | 0.178 |
| SN166Yb | syntaxin-1 | L | 205 | HD2* | 0.138 |
| SN166Yb | syntaxin-1 | I | 209 | HD1* | 0.217 |
| SN166Yb | syntaxin-1 | L | 212 | HD1* | 0.543 |
| SN166Yb | syntaxin-1 | L | 212 | HD2* | 0.516 |
| SN166Yb | syntaxin-1 | M | 215 | HE* | 0.310 |
| SN166Yb | syntaxin-1 | M | 217 | HE* | 0.120 |
| SN166Yb | syntaxin-1 | M | 219 | HE* | 0.446 |
| SN166Yb | syntaxin-1 | M | 221 | HE* | 0.054 |
| SN166Yb | syntaxin-1 | L | 222 | HD1* | 0.141 |
| SN166Yb | syntaxin-1 | L | 222 | HD2* | 0.090 |
| SN166Yb | syntaxin-1 | V | 223 | HG1* | -0.013 |
| SN166Yb | syntaxin-1 | V | 223 | HG2* | 0.164 |
| SN166Yb | syntaxin-1 | M | 229 | HE* | -0.021 |
| SN166Yb | syntaxin-1 | I | 230 | HD1* | -0.131 |
| SN166Yb | syntaxin-1 | I | 233 | HD1* | -0.066 |
| SN166Yb | syntaxin-1 | V | 237 | HG1* | -0.058 |
| SN166Yb | syntaxin-1 | V | 237 | HG2* | -0.058 |
| SN166Yb | syntaxin-1 | V | 241 | HG1* | -0.024 |
| SN166Yb | syntaxin-1 | V | 241 | HG2* | -0.041 |
| SN166Yb | syntaxin-1 | V | 244 | HG1* | -0.032 |
| SN166Yb | syntaxin-1 | V | 244 | HG2* | -0.027 |
| SN166Yb | syntaxin-1 | V | 248 | HG1* | -0.012 |
| SN166Yb | syntaxin-1 | V | 248 | HG2* | -0.018 |
| SN41Dy | SNC | M | 146 | CE | -0.164 |
| SN41Dy | SNC | M | 146 | HE* | -0.158 |
| SN41Dy | SNC | L | 150 | CD1 | -0.389 |
| SN41Dy | SNC | L | 150 | CD2 | -0.539 |
| SN41Dy | SNC | L | 150 | HD1* | -0.370 |
| SN41Dy | SNC | L | 150 | HD2* | -0.526 |
| SN41Dy | SNC | I | 157 | CD1 | -1.425 |
| SN41Dy | SNC | I | 157 | HD1* | -1.533 |
| SN41Dy | SNC | L | 165 | CD1 | -0.450 |
| SN41Dy | SNC | L | 165 | CD2 | -0.508 |
| SN41Dy | SNC | L | 165 | HD1* | -0.467 |
| SN41Dy | SNC | L | 165 | HD2* | -0.488 |
| SN41Dy | SNC | I | 171 | CD1 | -0.232 |
| SN41Dy | SNC | I | 171 | HD1* | -0.312 |
| SN41Dy | SNC | I | 181 | CD1 | 0.193 |
| SN41Dy | SNC | I | 181 | HD1* | 0.192 |
| SN41Dy | SNC | M | 182 | CE | 0.143 |
| SN41Dy | SNC | M | 182 | HE* | 0.138 |
| SN41Dy | SNC | I | 192 | CD1 | 0.067 |
| SN41Dy | SNC | I | 192 | HD1* | 0.082 |
| SN41Dy | SNC | L | 203 | CD1 | 0.019 |
| SN41Dy | SNC | L | 203 | CD2 | 0.015 |
| SN41Dy | SNC | L | 203 | HD1* | 0.021 |
| SN41Dy | SNC | L | 203 | HD2* | 0.010 |
| SN41Dy | synaptobrevin | L | 32 | CD1 | -0.382 |
| SN41Dy | synaptobrevin | L | 32 | HD1* | -0.382 |
| SN41Dy | synaptobrevin | V | 42 | CG2 | -1.126 |
| SN41Dy | synaptobrevin | V | 42 | HG2* | -1.207 |
| SN41Dy | synaptobrevin | Ile | 45 | CD1 | -0.721 |
| SN41Dy | synaptobrevin | Ile | 45 | HD1* | -0.723 |
| SN41Dy | synaptobrevin | Val | 48 | CG1 | -0.438 |
| SN41Dy | synaptobrevin | Val | 48 | CG2 | -0.480 |
| SN41Dy | synaptobrevin | Val | 48 | HG1* | -0.407 |
| SN41Dy | synaptobrevin | Val | 48 | HG2* | -0.470 |
| SN41Dy | synaptobrevin | V | 50 | CG1 | -0.673 |
| SN41Dy | synaptobrevin | V | 50 | HG1* | -0.721 |
| SN41Dy | synaptobrevin | V | 53 | CG1 | -0.151 |
| SN41Dy | synaptobrevin | V | 53 | HG1* | -0.077 |
| SN41Dy | synaptobrevin | L | 54 | CD2 | 0.144 |
| SN41Dy | synaptobrevin | L | 54 | HD2* | 0.171 |
| SN41Dy | synaptobrevin | L | 60 | CD2 | 0.342 |
| SN41Dy | synaptobrevin | L | 60 | HD2* | 0.393 |
| SN41Dy | synaptobrevin | L | 70 | CD1 | 0.095 |
| SN41Dy | synaptobrevin | L | 70 | HD1* | 0.109 |
| SN41Dy | synaptobrevin | L | 84 | CD1 | 0.025 |
| SN41Dy | synaptobrevin | L | 84 | CD2 | 0.023 |
| SN41Dy | synaptobrevin | L | 84 | HD1* | 0.036 |
| SN41Dy | synaptobrevin | L | 84 | HD2* | 0.026 |
| SN41Dy | syntaxin-1 | L | 192 | CD1 | -0.071 |
| SN41Dy | syntaxin-1 | L | 192 | CD2 | -0.103 |
| SN41Dy | syntaxin-1 | L | 192 | HD1* | -0.058 |
| SN41Dy | syntaxin-1 | L | 192 | HD2* | -0.055 |
| SN41Dy | syntaxin-1 | I | 203 | CD1 | -0.341 |
| SN41Dy | syntaxin-1 | I | 203 | HD1* | -0.327 |
| SN41Dy | syntaxin-1 | L | 205 | CD1 | -0.950 |
| SN41Dy | syntaxin-1 | L | 205 | CD2 | -0.765 |
| SN41Dy | syntaxin-1 | L | 205 | HD1* | -0.883 |
| SN41Dy | syntaxin-1 | L | 205 | HD2* | -0.705 |
| SN41Dy | syntaxin-1 | M | 215 | CE | -1.641 |
| SN41Dy | syntaxin-1 | M | 215 | HE* | -1.563 |
| SN41Dy | syntaxin-1 | M | 219 | CE | -0.939 |
| SN41Dy | syntaxin-1 | M | 219 | HE* | -0.857 |
| SN41Dy | syntaxin-1 | I | 230 | CD1 | 0.679 |
| SN41Dy | syntaxin-1 | I | 230 | HD1* | 0.614 |
| SN41Dy | syntaxin-1 | I | 233 | CD1 | 0.367 |
| SN41Dy | syntaxin-1 | I | 233 | HD1* | 0.364 |
| SN41Dy | syntaxin-1 | V | 237 | CG1 | 0.112 |
| SN41Dy | syntaxin-1 | V | 237 | CG2 | 0.163 |
| SN41Dy | syntaxin-1 | V | 237 | HG1* | 0.156 |
| SN41Dy | syntaxin-1 | V | 237 | HG2* | 0.176 |
| SN41Dy | syntaxin-1 | V | 241 | CG1 | 0.049 |
| SN41Dy | syntaxin-1 | V | 241 | CG2 | 0.077 |
| SN41Dy | syntaxin-1 | V | 241 | HG1* | 0.063 |
| SN41Dy | syntaxin-1 | V | 241 | HG2* | 0.111 |
| SN41Dy | syntaxin-1 | V | 244 | CG1 | 0.068 |
| SN41Dy | syntaxin-1 | V | 244 | CG2 | 0.071 |
| SN41Dy | syntaxin-1 | V | 244 | HG1* | 0.091 |
| SN41Dy | syntaxin-1 | V | 244 | HG2* | 0.073 |
| SN41Dy | syntaxin-1 | V | 248 | CG1 | 0.053 |
| SN41Dy | syntaxin-1 | V | 248 | CG2 | 0.079 |
| SN41Dy | syntaxin-1 | V | 248 | HG1* | 0.041 |
| SN41Dy | syntaxin-1 | V | 248 | HG2* | 0.051 |
| Syx214Dy | SNC | M | 146 | CE | -0.133 |
| Syx214Dy | SNC | M | 146 | HE* | -0.139 |
| Syx214Dy | SNC | L | 150 | CD1 | -0.385 |
| Syx214Dy | SNC | L | 150 | CD2 | -0.479 |
| Syx214Dy | SNC | L | 150 | HD1* | -0.340 |
| Syx214Dy | SNC | L | 150 | HD2* | -0.464 |
| Syx214Dy | SNC | V | 153 | CG1 | -0.904 |
| Syx214Dy | SNC | V | 153 | CG2 | -0.739 |
| Syx214Dy | SNC | V | 153 | HG1* | -0.904 |
| Syx214Dy | SNC | V | 153 | HG2* | -0.678 |
| Syx214Dy | SNC | I | 156 | CD1 | -0.695 |
| Syx214Dy | SNC | I | 156 | HD1* | -0.709 |
| Syx214Dy | SNC | I | 157 | CD1 | -1.416 |
| Syx214Dy | SNC | I | 157 | HD1* | -1.361 |
| Syx214Dy | SNC | M | 163 | CE | -0.922 |
| Syx214Dy | SNC | M | 163 | HE* | -0.885 |
| Syx214Dy | SNC | L | 165 | CD1 | -0.444 |
| Syx214Dy | SNC | L | 165 | CD2 | -0.427 |
| Syx214Dy | SNC | L | 165 | HD1* | -0.433 |
| Syx214Dy | SNC | L | 165 | HD2* | -0.399 |
| Syx214Dy | SNC | I | 178 | CD1 | 0.455 |
| Syx214Dy | SNC | I | 178 | HD1* | 0.492 |
| Syx214Dy | SNC | I | 181 | CD1 | 0.231 |
| Syx214Dy | SNC | I | 181 | HD1* | 0.229 |
| Syx214Dy | SNC | M | 182 | CE | 0.352 |
| Syx214Dy | SNC | M | 182 | HE* | 0.362 |
| Syx214Dy | SNC | I | 192 | CD1 | 0.100 |
| Syx214Dy | SNC | I | 192 | HD1* | 0.100 |
| Syx214Dy | SNC | M | 202 | CE | 0.036 |
| Syx214Dy | SNC | M | 202 | HE* | 0.027 |
| Syx214Dy | SNC | L | 203 | CD1 | 0.026 |
| Syx214Dy | SNC | L | 203 | CD2 | 0.022 |
| Syx214Dy | SNC | L | 203 | HD1* | 0.036 |
| Syx214Dy | SNC | L | 203 | HD2* | 0.030 |
| Syx214Dy | SNN | L | 11 | CD1 | 0.010 |
| Syx214Dy | SNN | L | 11 | CD2 | 0.028 |
| Syx214Dy | SNN | L | 11 | HD1* | 0.018 |
| Syx214Dy | SNN | L | 11 | HD2* | 0.019 |
| Syx214Dy | SNN | M | 14 | CE | 0.039 |
| Syx214Dy | SNN | M | 14 | HE* | 0.031 |
| Syx214Dy | SNN | L | 21 | CD1 | 0.075 |
| Syx214Dy | SNN | L | 21 | CD2 | 0.057 |
| Syx214Dy | SNN | L | 21 | HD1* | 0.056 |
| Syx214Dy | SNN | L | 21 | HD2* | 0.084 |
| Syx214Dy | SNN | L | 26 | CD1 | -0.164 |
| Syx214Dy | SNN | L | 26 | CD2 | -0.139 |
| Syx214Dy | SNN | L | 26 | HD1* | -0.161 |
| Syx214Dy | SNN | L | 26 | HD2* | -0.105 |
| Syx214Dy | SNN | M | 32 | CE | -0.553 |
| Syx214Dy | SNN | M | 32 | HE* | -0.540 |
| Syx214Dy | SNN | V | 36 | CG1 | -1.404 |
| Syx214Dy | SNN | V | 36 | CG2 | -1.138 |
| Syx214Dy | SNN | V | 36 | HG1* | -1.408 |
| Syx214Dy | SNN | V | 36 | HG2* | -1.028 |
| Syx214Dy | SNN | I | 44 | CD1 | -0.631 |
| Syx214Dy | SNN | I | 44 | HD1* | -0.603 |
| Syx214Dy | SNN | L | 47 | CD1 | -0.497 |
| Syx214Dy | SNN | L | 47 | CD2 | -0.508 |
| Syx214Dy | SNN | L | 47 | HD1* | -0.487 |
| Syx214Dy | SNN | L | 47 | HD2* | -0.484 |
| Syx214Dy | SNN | V | 48 | CG1 | -0.215 |
| Syx214Dy | SNN | V | 48 | CG2 | -0.318 |
| Syx214Dy | SNN | V | 48 | HG1* | -0.194 |
| Syx214Dy | SNN | V | 48 | HG2* | -0.316 |
| Syx214Dy | SNN | L | 50 | CD1 | -0.134 |
| Syx214Dy | SNN | L | 50 | CD2 | 0.200 |
| Syx214Dy | SNN | L | 50 | HD1* | -0.110 |
| Syx214Dy | SNN | L | 50 | HD2* | 0.161 |
| Syx214Dy | SNN | L | 57 | CD1 | 0.273 |
| Syx214Dy | SNN | L | 57 | CD2 | 0.301 |
| Syx214Dy | SNN | L | 57 | HD1* | 0.272 |
| Syx214Dy | SNN | L | 57 | HD2* | 0.284 |
| Syx214Dy | SNN | V | 60 | CG1 | 0.184 |
| Syx214Dy | SNN | V | 60 | CG2 | 0.158 |
| Syx214Dy | SNN | V | 60 | HG1* | 0.196 |
| Syx214Dy | SNN | V | 60 | HG2* | 0.186 |
| Syx214Dy | SNN | M | 64 | CE | 0.154 |
| Syx214Dy | SNN | M | 64 | HE* | 0.169 |
| Syx214Dy | SNN | I | 67 | CD1 | 0.098 |
| Syx214Dy | SNN | I | 67 | HD1* | 0.112 |
| Syx214Dy | SNN | M | 71 | CE | 0.043 |
| Syx214Dy | SNN | M | 71 | HE* | 0.071 |
| Syx214Dy | SNN | L | 78 | CD1 | 0.067 |
| Syx214Dy | SNN | L | 78 | CD2 | 0.051 |
| Syx214Dy | SNN | L | 78 | HD1* | 0.038 |
| Syx214Dy | SNN | L | 78 | HD2* | 0.034 |
| Syx214Dy | SNN | L | 81 | CD1 | 0.053 |
| Syx214Dy | SNN | L | 81 | CD2 | 0.098 |
| Syx214Dy | SNN | L | 81 | HD1* | 0.046 |
| Syx214Dy | SNN | L | 81 | HD2* | 0.039 |

______________________________________________________________________________

^*^ The estimated uncertainty is 0.008 ppm for PCSs measured on ^1^H chemical shifts and 0.05 ppm for PCSs measured on ^13^C chemical shifts.
