# Supplementary material for: Sequence-specific assignment of methyl groups from the neuronal SNARE complex using lanthanide-induced pseudocontact shifts
Source: J Biomol NMR. 2016 Dec 17;66(4):281–93. doi: 10.1007/s10858-016-0078-1 (PMC5216067; doi:10.1007/s10858-016-0078-1)
Supplement: Supplementary file 2 — Supplementary material 2 (PDF 473 KB) [file 10858_2016_78_MOESM2_ESM.pdf]

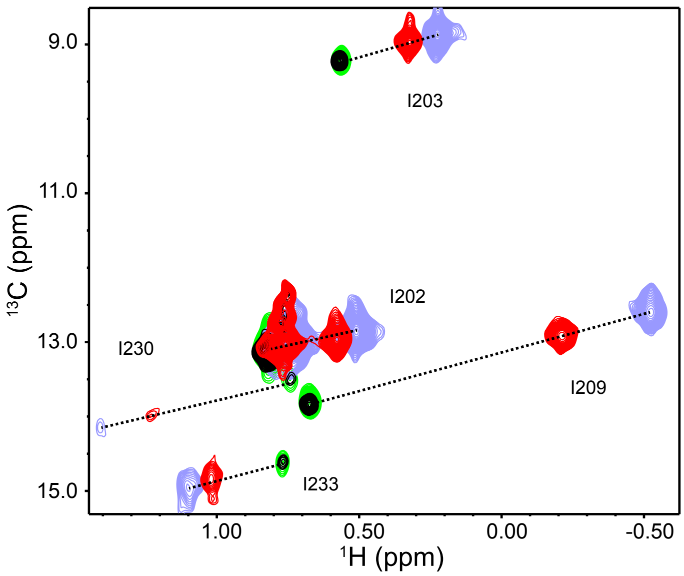

Supplementary Figure 1. KSCN alters the PCSs induced in the SNARE complex. The superimposed contour plots show expansions of the Ile-δ1 region of ^{1}H-^{13}C HMQC spectra of SNARE complexes containing ^{2}H,$^{13}CH_3$-labeled syntaxin-1 that were untagged (black and green contours) or tagged at residue 166 of SNAP-25 with Dy^{3+}-loaded C2 (red and violet contours). The spectra shown in red and black contours were acquired in the standard buffer used in this study (20 mM Tris pH 7.4, 200 mM NaCl) whereas the spectra shown in green and violet contours were acquired in the buffer used in Brewer et al. 2015 (25 mM Tris pH 7.4, 125 mM KSCN). The PCSs caused on the Ile cross-peaks are illustrated by dashed lines and the corresponding cross-peak assignments are indicated.
